# Dose-dependent effects of necrostatin-1 supplementation to tissue culture media of young porcine islets

**DOI:** 10.1371/journal.pone.0243506

**Published:** 2020-12-07

**Authors:** Hien Lau, Nicole Corrales, Samuel Rodriguez, Colleen Luong, Mohammadreza Mohammadi, Veria Khosrawipour, Shiri Li, Michael Alexander, Paul de Vos, Jonathan R. T. Lakey

**Affiliations:** 1 Department of Surgery, University of California Irvine, Irvine, California, United States of America; 2 Department of Materials Science and Engineering, Sue and Bill Gross Stem Cell Research Center, University of California Irvine, Irvine, California, United States of America; 3 Department of Biomedical Engineering, University of California Irvine, Irvine, California, United States of America; 4 Immunoendocrinology, Department of Pathology and Medical Biology, University of Groningen, Groningen, The Netherlands; University of Szeged, HUNGARY

## Abstract

Previous studies have shown that necrostatin-1 (Nec-1) supplementation improved the viability of murine islets following exposure to nitric oxide, increased the survival of human islets during hypoxic culture, and augmented the maturation of pre-weaned porcine islets (PPIs) after 7 days of tissue culture. A limitation of these studies is that only one concentration of Nec-1 was used, and no studies have determined the optimal dose of Nec-1 for PPIs. Thus, the present study examined the effects of Nec-1 on PPIs at four different doses—0, 25, 50, 100, and 200 μM—after 7 days of tissue culture when supplemented on day 3. PPIs were isolated from pancreata of pre-weaned Yorkshire piglets (8–15 days old) and cultured in a specific islet maturation media added with Nec-1 on day 3 of tissue culture at 4 different doses—0, 25, 50, 100, and 200 μM (n = 6 for each dose). After 7 days of tissue culture, islets were assessed for recovery, viability, endocrine cellular content, GLUT2 expression in beta cells, and insulin secretion after glucose challenge. Nec-1 did not affect the viability of both intact islets and dissociated islets cells during tissue culture regardless of doses. Islets cultured in media supplemented with Nec-1 at 100 μM, but not 25, 50, or 200 μM, had a significantly higher recovery, composition of endocrine cells, GLUT2 expression in beta cells, and insulin secretion capacity than control islets cultured in media without Nec-1 supplementation. Moreover, culturing islets in 200 μM Nec-1 supplemented media not only failed to improve the insulin release but resulted in a lower glucose-induced insulin stimulation index compared to islets cultured in media added with 100 μM Nec-1. Xenotransplantation using porcine islets continues to demonstrate scientific advances to justify this area of research. Our findings indicate that Nec-1 supplementation at 100 μM was most effective to enhance the *in vitro* maturation of PPIs during tissue culture.

## Introduction

Type 1 diabetes mellitus (T1DM) is an autoimmune disease characterized by the gradual destruction of insulin-secreting beta cells in pancreatic islets [1]. As a consequence of insulin deficiency, patients develop hyperglycemia and are increasingly susceptible to ketoacidosis [2, 3]. The conventional treatment for T1DM involves daily subcutaneous injections of insulin [4]. Although this therapy can manage the disease, the increased risk of hypoglycemic episodes, treatment’s inducement of pain, and requirement of multiple doses a day can deter patients from utilizing the treatment correctly [5, 6]. In the search for an alternative treatment for T1DM, islet allotransplantation has been shown as a potential therapy to restore glucose homeostasis in patients with T1DM [7]. Currently, the pancreata of cadaveric donors are the source of islets for the majority of islet transplants [8]. However, the universal implementation of islet allotransplantation has been hindered by the limited availability of suitable cadaveric donors and high-quality viable human islets [9].

To address the scarcity of human donor pancreata, intensive research has identified porcine pancreata as suitable donors for islets [10]. Porcine pancreata serve as a competent source for islet xenotransplantation as they are able to offer a potential unlimited supply of islets [11]. Another advantage involves the structural resemblance of insulin in pigs and humans with a variation of only one amino acid [12, 13]. Young porcine islets isolated from either neonatal or pre-weaned juvenile porcine pancreata have shown potential in terms of cost-effectiveness and functionality [14, 15]. Despite these advantages, young porcine islets require prolonged culture to reach optimal maturation before transplantation [16]. As a substantial loss of islets has been reported during this *in vitro* tissue culture period, the use of growth factors could assist to shorten culture time and accelerate islet development [16–19].

Necrostatin-1 (Nec-1) is a specific allosteric inhibitor of receptor-interacting protein-1 (RIP1) kinase, a downstream signaling molecule of the necroptosis pathway [20]. As a result, Nec-1 has been demonstrated to block necroptosis and improve cell survival in various animal models of ischemic injury [20–24]. Emerging data has also shown the effects of Nec-1 at different doses on various cell types. In a study that examined the effects of Nec-1 concentrations ranging from 1 μM to 100 μM on murine L929SA cells, a higher dose of Nec-1 was more effective at reducing RIP1 kinase autophosphorylation and necroptosis [25]. This dose-dependent effect of Nec-1 has also been confirmed in recombinant human RIP1 kinase [25]. Moreover, 10 μM of Nec-1 was enough to inhibit TNF-induced necroptosis in a murine fibrosarcoma cell line [25]. In explanted outer hair cells of adult CBA/J mice, a Nec-1 dose of up to 300 could be used without marked damage [26]. Despite its potential, the effects of Nec-1 on islets has not been fully elucidated [27]. In beta-cell lines and mouse islets, Nec-1 could inhibit cell death after nitric oxide exposure [28]. Moreover, Nec-1 decreased the S-nitrosoglutathione (GSNO)-induced release of high mobility group box 1 (HMGB1) and cyclophilin A, both of which account for enhancing islet inflammation [28]. Another study has demonstrated the effects of Nec-1 on encapsulated human islets that were cultured in low-nutrient and hypoxic conditions [29]. Assessments after 7 days of tissue culture revealed that Nec-1 inhibited the release of danger-associated molecular patterns (DAMPs), dsDNA, and uric acid [29]. In regards to young porcine islets, our lab has recently reported that Nec-1 supplementation to islet tissue culture media significantly enhanced islet insulin content, development of endocrine cells, GLUT2 expression in beta-cells, and insulin secretion [17]. Furthermore, the addition of Nec-1 to tissue culture media on day 3 rather than immediately after islet isolation has been shown to improve the recovery and glucose-induced insulin stimulation index of pre-weaned porcine islets (PPIs) [30]. As the optimal activity level of Nec-1 has been shown to depend both on the concentration and the cell type, the use of only one dose of Nec-1 in previous islet studies represents a major limitation. Due to the variations in concentrations of Nec-1 that have been used for different cell types, optimizing the dose of Nec-1 for PPIs may assist to improve its previously established benefits. Therefore, the present study examined the effects of Nec-1 on PPIs at four different doses (25, 50, 100, and 200 μM) after 7 days of tissue culture when supplemented on day 3.

## Material and methods

### PPI isolation

Animal procedures were authorized by the Institutional Animal Care and Use Committee (IACUC) at the University of California, Irvine. Pancreata from 8-to-15-day-old, pre-weaned Yorkshire pigs were procured for islet isolation [31]. All surgery and euthanasia were performed at University of California, Irvine. Euthanasia was done by cardiac puncture and exsanguination under anesthesia by IACUC approved personnel. Following procurement (<5 minutes), pancreata were placed in cold HBSS. The cold ischemic time was restricted to under 1 hour. Pancreata were then minced into 1mm^3^ tissue fragments and digested with Sigma Type V Collagenase (2.5 mg/mL, dissolved in HBSS; cat#C8051, Sigma Aldrich) in a 37°C, 100 rpm shaking water bath for 15 minutes. Enzymatic digestion was quenched with HBSS supplemented with 1% porcine serum solution, and the digested tissue was filtered through 500 μm stainless steel mesh.

### Islet culture and Nec-1 treatment

Isolated islets were cultured in an islet maturation culture media supplemented with 50% Ham’s F-12 medium (cat# 10–080, Corning Inc.), 50% medium 199 (cat# 50-051-PB, Corning Inc.), 10 mM HEPES (cat# H3375, Sigma-Aldrich), 5 mM L-glutathione (cat# G4251, Sigma-Aldrich), 0.6 mL/L ITS+3 (cat# I2771, Sigma-Aldrich), 10 mM nicotinamide (cat# N5535, Sigma-Aldrich), 100 ug/mL gentamicin sulfate (cat# 30-005-CR, Corning Inc.), 10 uM trolox (cat# 238813, Sigma-Aldrich), 200 U/L heparin (cat# 400–10, Sagent Pharmaceuticals), 0.1 mM pefabloc (cat# sc202041B, Santa Cruz Biotechnology), 2 mM L-glutamine (cat# 56-85-9, Alfa Aesar), 2.5 mM calcium chloride dihydrate (cat#C79-3, Fisher Scientific), 1000 U/L DNase (cat#D4263, Sigma-Aldrich), antibiotic/antimycotic solution (Corning Inc, cat#30004CI), and 10% porcine serum using T-150 untreated suspension flasks (cat #CLS430825, Corning Inc) for 7 days in a 37°C, 5% CO^2^ humidified incubator (cat#3110, Thermo Forma Series II 3120 Water Jacketed Incubators) [19]. A full media change was performed 24 hours post islet isolation, and a half media change was done every 48 hours thereafter. On day 3 of tissue culture, Nec-1 (Abcam, cat#ab141053) was supplemented to islet tissue culture media at four different doses: 25, 50, 100, and 200 μM (n = 6 each) [30]. Islets cultured in media without Nec-1 supplementation served as the control group (n = 6).

### Islet recovery

Islet equivalence (IEQ) was acquired by staining 100 μL islet aliquot with 1 mL of dithizone (DTZ, MP Biomedicals, cat#150999) for 5 minutes and counting stained islets on a standard stereo microscope (Max Erb) with a 10× eyepiece graticule [31, 32]. The percentage of IEQ on day 7 of tissue culture normalized to day 3 of tissue culture before Nec-1 supplementation was calculated to obtain the islet recovery [17].

### Islet viability

On day 7 of tissue culture, 100 IEQ were stained for 30 minutes at room temperature with Calcein AM (CalAM, Invitrogen, cat#C1430) to detect live islets and propidium iodide (PI, Invitrogen, cat#P3566) to detect dead or dying islets [17]. Stained islets were evaluated on a microplate reader (Tecan Infinite F200; Tecan) and the viability was calculated by the following equation:

CalAM−positivecells/(CalAM−positivecells+PI−positivecells)×100.

### Islet cellular viability, endocrine cellular composition, and GLUT2 expression in beta cells

Accutase (cat#AT104-500, Innovative Cell Technologies) was used on day 7 of tissue culture to dissociate 3000 IEQ for 15 minutes in a 37°C, 100 rpm shaking water bath [33]. The islets were filtered through a 40 μm filter (VMR) to acquire a single-cell suspension. Samples were then stained with 7-aminoactinomycin D viability dye (7-AAD; cat#A1310, Invitrogen) for 30 minutes at 4°C to identify live and dead cells. Following staining, cells were fixed with 4% paraformaldehyde for 10 minutes and permeabilized with Intracellular Staining Permeabilization Wash Buffer (cat#421002, BioLegend) on ice for 15 minutes. Cells were incubated with Protein Block (cat#ab64226, Abcam) on ice for 30 minutes to decrease non-specific binding. After blocking, cells were stained with fluorescently conjugated antibodies in Intracellular Staining Permeabilization Wash Buffer (cat#421002, BioLegend) supplemented with 0.5% bovine serum albumin (BSA; cat#BAL62-0500, Equitech-Bio, Inc) on ice for 30 minutes. Beta cells, alpha cells, and delta cells were identified by PE-conjugated anti-insulin (cat#8508, CST), APC-conjugated anti-glucagon (cat#NBP2-21803AF647, Novus Biological), and Alexa Fluor 488-conjugated anti-somatostatin (cat#566032, BD Biosciences) antibodies, respectively. Double staining with FITC-conjugated anti-GLUT2 (cat#FAB1414G-100UG, Novus Biological) antibody and PE-conjugated anti-insulin antibody were performed to detect GLUT2-positive beta cells. All cell samples were analyzed on the NovoCyte 3000VYB Flow Cytometer (ACEA Biosciences, Inc). FlowJo software (FlowJo, Ashland, OR) was utilized to further quantify cell populations. Unstained, single-stained, fluorescence minus one, and matching isotype control samples were used as gating controls.

### Islet function

Glucose-stimulated insulin release (GSIR) assay was performed on day 7 of tissue culture to evaluate *in vitro* islet secretion in response to glucose challenge [31]. For each sample, sets of three 100 IEQ were incubated at 37°C and 5% CO^2^ in the following order of glucose media for 1 hour each: low glucose (2.8 mM; L1), high glucose (28 mM; H), low glucose (2.8 mM; L2), and high glucose plus 3-isobutyl-1-methylxanthine (28 mM + 0.1 mM IBMX; H+). The supernatant was collected after every hour for storage at -20°C until assessment. Insulin concentration in the supernatant was measured with a porcine insulin enzyme-linked immunosorbent assay kit (Porcine Insulin ELISA; cat#10-1200-01, Mercodia) and analyzed on a microplate reader (Infinite F200, Tecan and Magellan V7). The insulin concentration was normalized to the DNA content in each sample and expressed as pg of insulin/ng of DNA. The islet insulin secretion in high glucose media was divided by the insulin secreted in the first low glucose media to obtain the glucose-induced insulin stimulation index (SI).

### Islet DNA content

Islets were lysed with cell lysis buffer (10 mM Tris-HCl, 1 mM EDTA, 1% Triton X-100, pH 8) and sonicated (Sonics VibraCell Ultrasonic Processor Model VC70T, Sonics & Materials, Inc) for 30 seconds on ice [34]. Samples were then centrifuged at 1400 g and 4°C for 15 minutes. The supernatant was collected, and its DNA content was stained with a fluorescent DNA dye (Quant-iT PicoGreen dsDNA kit, cat #Q32850, Molecular Probes) and analyzed on a microplate reader (Infinite F200, Tecan and Magellan V7).

### Statistical analysis

Data normality was examined using a Shapiro-Wilk test. Data with a normal distribution are expressed as mean ± standard error of the mean (SEM), while data with a non-normal distribution are presented median and interquartile range. Statistical significance was computed by either a one-way ANOVA test followed by a post hoc Tukey’s HSD test for normally distributed data or a Kruskal-Wallis test followed by a post hoc Dunn’s Multiple Comparison test for non-normally distributed data. P-values < .05 were considered statistically significant. GraphPad Prism (GraphPad Software 8.0.1) was used to analyze data.

## Results

### 100 μM Nec-1 improves the recovery of PPIs

**Fig 1:**
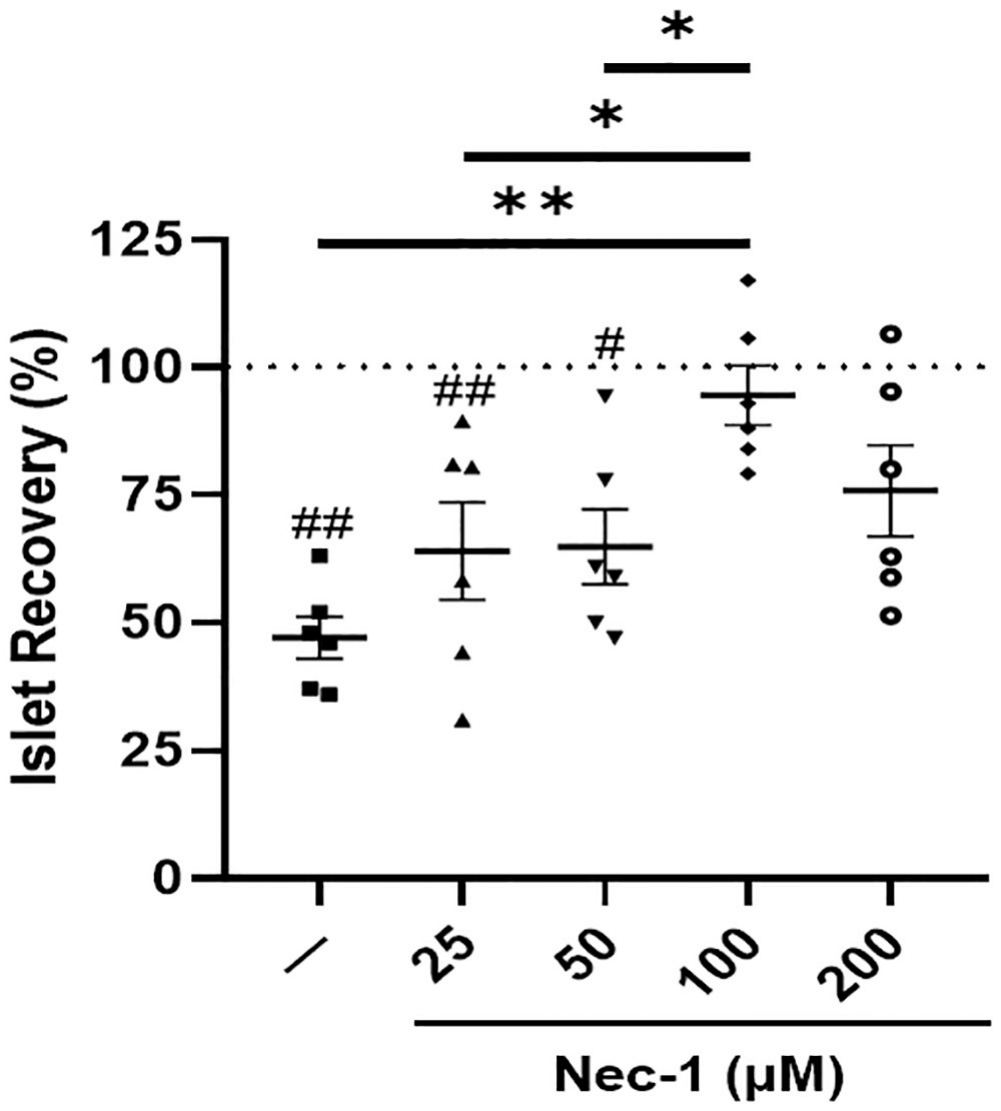
Islet recovery after 7 days of tissue culture in control media (—) or media supplemented with 4 different doses of necrostatin-1 (25, 50, 100, or 200 μM of Nec-1) on day 3. Islet recovery was calculated by normalizing the islet equivalents of all groups on day 7 to day 3 before Nec-1 supplementation (dotted line) and expressed as percentage. n = 6 for each group. ^#^p < .05 vs day 3. ^##^p < .01 vs day 3. *p < .05. **p < .01. Data are expressed as mean ± SEM.

The effects of Nec-1 on the recovery of PPIs when added on day 3 were examined on day 7 of tissue culture at 4 different doses—25, 50, 100, and 200 μM. Without Nec-1 supplementation, there was a significant loss of islets (47.1 ± 4.1%) on day 7 of tissue culture (p < .01, Fig 1). However, culturing islets in media supplemented with Nec-1 at either 100 or 200 μM, but not at 25 or 50 μM, (25 μM: 64.1 ± 9.5%, 50 μM: 64.9 ± 7.4%, 100 μM: 94.5 ± 5.8%, and 200 μM: 75.9 ± 8.9%) prevented the loss of islet mass on day 7 of tissue culture compared to day 3 before adding Nec-1 (p = .99, p = .15, p < .01, and p < .05, respectively, Fig 1). Moreover, the recovery of islets cultured in media supplemented with 100 μM Nec-1 was significantly higher than control islets on day 7 of tissue culture (p < .01, Fig 1). This effect was not observed in islets cultured in 25, 50, or 200 μM Nec-1 supplemented media (p = .50, p = .45, and p = .06, respectively, Fig 1). Islets cultured in media added with 100 μM Nec-1 also had a significantly higher recovery than islets cultured in media added with 25 or 50 μM Nec-1 (p < .05, p < .05, respectively, Fig 1). Raising the dose of Nec-1 to 200 μM did not result in higher islet recovery compared to adding Nec-1 at three lower doses—25, 50, or 100 μM (p = .82, p = .86, and p = .40, respectively, Fig 1).

### The viability of PPIs was unaffected by the supplementation of Nec-1 at different doses

**Fig 2:**
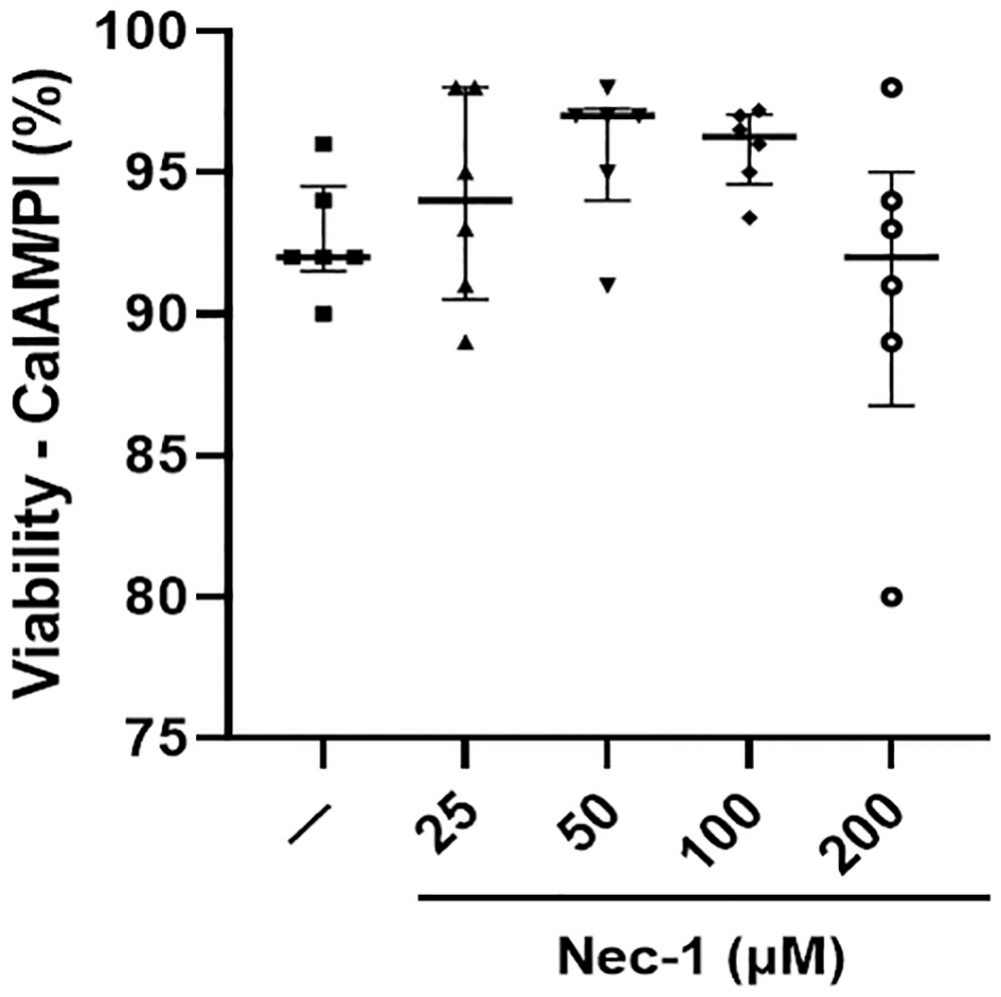
Islet viability after 7 days of tissue culture in control media (—) or media supplemented with 4 different doses of necrostatin-1 (25, 50, 100, or 200 μM of Nec-1) on day 3. 100 islet equivalents were incubated in Calcein AM (CalAM) and propidium iodide (PI) viability dyes for 30 minutes at room temperature and quantified on a microplate reader to analyze for live and dead cells, respectively. Islet viability was then calculated and expressed as percentage using the following equation: CalAM-positive cells/(CalAM-positive cells + PI-positive cells). n = 6 for each group. Data are expressed as median and interquartile range.

Islets were stained with CalAM/PI viability dye to determine the effects of Nec-1 supplementation at different doses on islet cell death. The addition of all four different doses of Nec-1 (25 μM: 94.0% [90.5, 98.0], 50 μM: 97.0% [94.0, 97.3], 100 μM: 96.3% [94.6, 97.1], and 200 μM: 92.0 [86.8, 95.0]) to the islet tissue culture media did not affect the islet viability compared to control islets on day 7 of tissue culture (92.0% [91.5, 94.5]) (p = .99, p = .61, p = .70, and p = .99, respectively, Fig 2).

**Fig 3:**
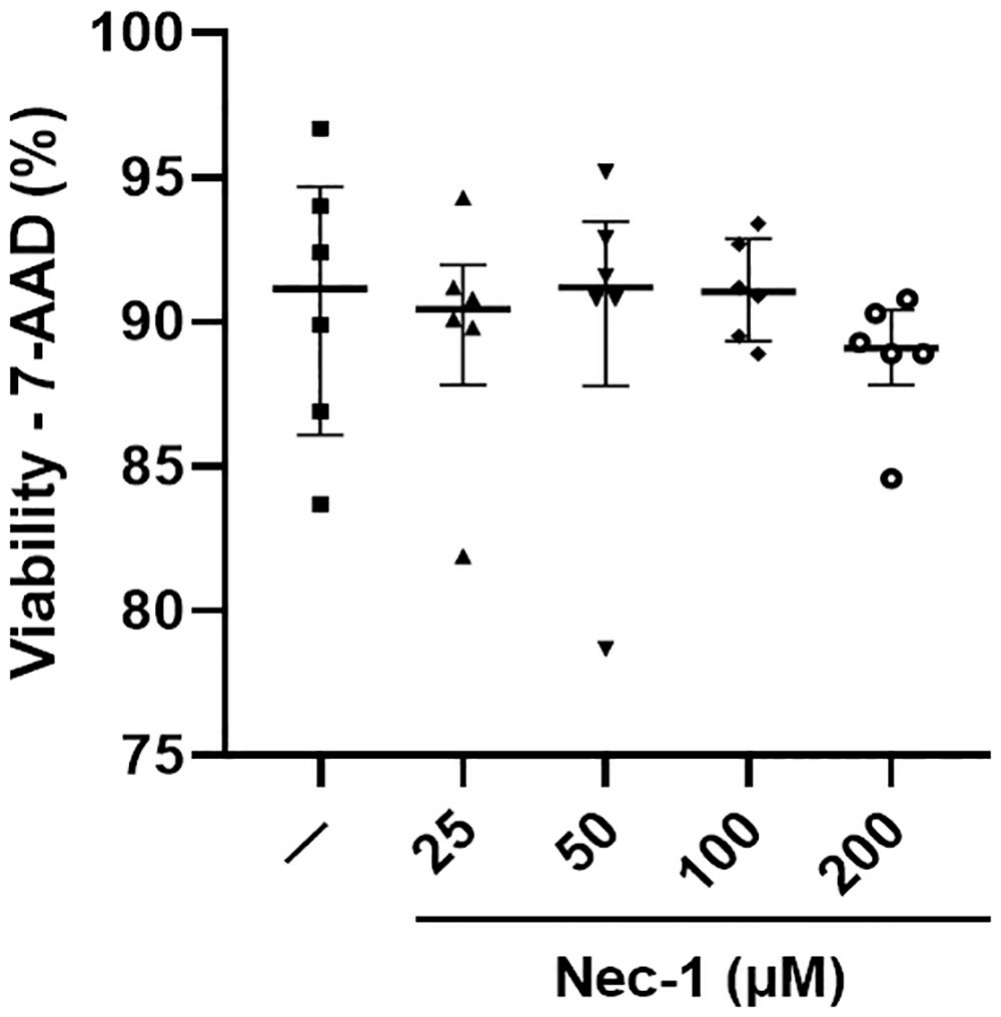
Viability of dissociated islet cells after 7 days of tissue culture in control media (—) or media supplemented with 4 different doses of necrostatin-1 (25, 50, 100, or 200 μM of Nec-1) on day 3. 3000 islet equivalents were dissociated into single cells using Accutase, stained with 7-amino-actinomycin D (7-AAD) viability dye for 30 minutes at 4°C, and analyzed by flow cytometry to quantify dead cells. n = 6 for each group. Data are expressed as median and interquartile range.

Since the pancreatic islet is a multicellular spherical system, assessing islet viability by CalAM/PI staining of intact islets alone may not have enough sensitivity to detect cell death in the islet core. Thus, intact islets were dissociated into single cells using Accutase, stained with 7-AAD viability dye, and analyzed by flow cytometry. The viability of dissociated islet cells was similar between control islets (91.2% [86.1, 94.7]) and islets cultured in media supplemented with 25, 50, 100, and 200 μM of Nec-1 on day 7 of tissue culture (90.5% [87.8, 92.0], 91.2% [87.8, 93.5], 91.1% [89.4, 92.9], and 89.1% [87.8, 90.4], respectively) (p = .99 for all four different doses of Nec-1, Fig 3).

### 100 μM Nec-1 increases the endocrine cellular composition of PPIs

**Fig 4:**
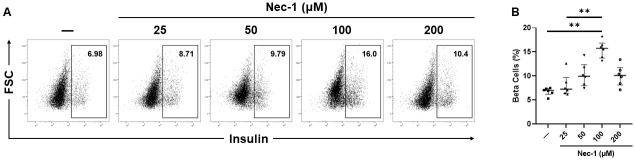
Beta-cell composition of islets after 7 days of tissue culture in control media (—) or media supplemented with 4 different doses of necrostatin-1 (25, 50, 100, or 200 μM of Nec-1) on day 3. 3000 islet equivalents were dissociated into single cells using Accutase, stained with 7-amino-actinomycin D (7-AAD) viability dye and phycoerythrin-conjugated anti-insulin antibody, and analyzed by flow cytometry to quantify the proportion of beta cells in islets. Insulin-positive beta cells were gated on 7-AAD-negative live cells. A) Representative flow cytometry plots of insulin staining. B) Percentage of insulin-positive beta cells. n = 6 for each group. **p < .01. Data are expressed as median and interquartile range.

After 7 days of tissue culture, the supplementation of Nec-1 at 100 μM, but not at 25, 50, or 200 μM, to the islet tissue culture media on day 3 (15.8% [13.7, 16.9], 7.3% [6.4, 9.7], 9.9% [8.0, 12.4], and 10.1% [8.1, 11.8], respectively) significantly increased the proportion of beta cells compared to control islets cultured in media without Nec-1 supplementation (6.9% [6.2, 7.4]) (p < .01, p = .99, p = .23, and p = .32, respectively, Fig 4A and 4B). Islets cultured in media supplemented with 25 μM Nec-1 also had a significantly lower beta-cell content than islets cultured in media with 100 μM Nec-1 supplementation (p < .01, Fig 4A and 4B).

**Fig 5:**
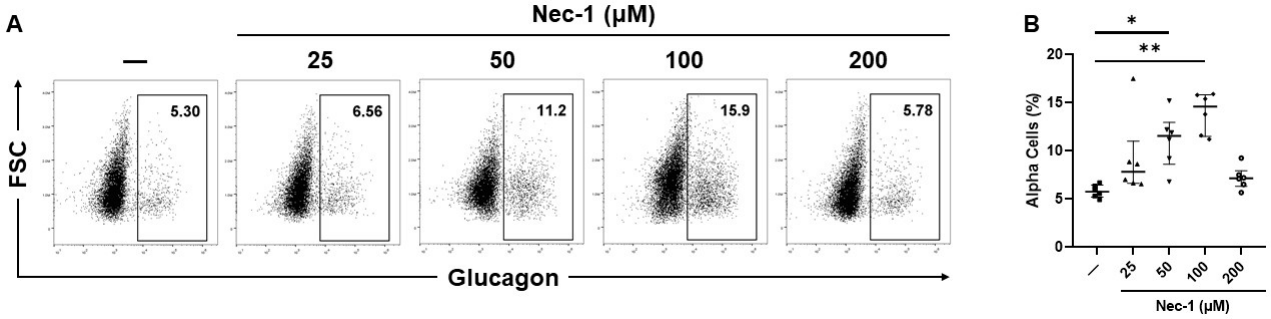
Alpha-cell composition of islets after 7 days of tissue culture in control media (—) or media supplemented with 4 different doses of necrostatin-1 (25, 50, 100, or 200 μM of Nec-1) on day 3. 3000 islet equivalents were dissociated into single cells using Accutase, stained with 7-amino-actinomycin D (7-AAD) viability dye and allophycocyanin-conjugated anti-glucagon antibody, and analyzed by flow cytometry to quantify the proportion of alpha cells in islets. Glucagon-positive alpha cells were gated on 7-AAD-negative live cells. A) Representative flow cytometry plots of glucagon staining. B) Percentage of glucagon-positive alpha cells. n = 6 for each group. *p < .05. **p < .01. Data are expressed as median and interquartile range.

Only the addition of 50 or 100 μM Nec-1, but not 25 or 200 μM Nec-1, to the islet tissue culture media (11.6% [8.6, 12.9], 14.6% [11.5, 15.8], 7.8% [6.6, 11.0], and 7.2% [6.3, 7.9], respectively) led to a significant increase in the alpha-cell content compared to control islets on day 7 of tissue culture (5.8% [5.2, 6.4]) (p < .05, p < .01, p = .33, and p = .99, respectively, Fig 5A and 5B).

**Fig 6:**
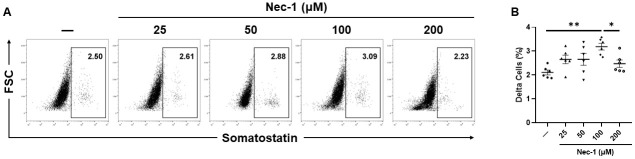
Delta-cell composition of islets after 7 days of tissue culture in control media (—) or media supplemented with 4 different doses of necrostatin-1 (25, 50, 100, or 200 μM of Nec-1) on day 3. 3000 islet equivalents were dissociated into single cells using Accutase, stained with 7-amino-actinomycin D (7-AAD) viability dye and fluorescein-isothiocyanate-conjugated anti-somatostatin antibody, and analyzed by flow cytometry to quantify the proportion of delta cells in islets. Somatostatin-positive delta cells were gated on 7-AAD-negative live cells. A) Representative flow cytometry plots of somatostatin staining. B) Percentage of somatostatin-positive delta cells. n = 6 for each group. *p < .05. **p < .01. Data are expressed as mean ± SEM.

While the delta-cell content of islets cultured in media supplemented with either 25, 50, or 200 μM Nec-1 (2.7 ± .2%, 2.7 ± .2%, and 2.5 ± .2%, respectively) was similar to control islets on day 7 of tissue culture (2.1 ± .1%), islets cultured in 100 μM Nec-1 supplemented media (3.2 ± .1%) had a 1.5 times increase in the proportion of delta cells compared to islets cultured in media without Nec-1 supplementation (p = .19, p = .19, p = .56, and p < .01, respectively, Fig 6A and 6B). Moreover, the proportion of delta cells in islets cultured in media supplemented with 100 μM Nec-1 was significantly higher than islets cultured in 200 μM Nec-1 supplemented media (p < .05, Fig 6A and 6B).

### 100 μM Nec-1 enhances GLUT2 expression in beta cells of PPIs

**Fig 7:**
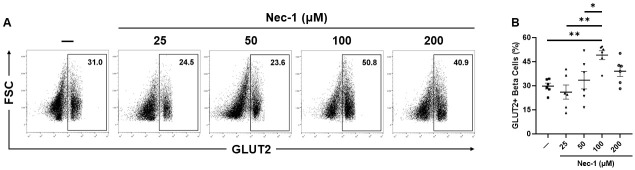
GLUT2 expression in beta cells of islets after 7 days of tissue culture in control media (—) or media supplemented with 4 different doses of necrostatin-1 (25, 50, 100, or 200 μM of Nec-1) on day 3. 3000 islet equivalents were dissociated into single cells using Accutase, stained with 7-amino-actinomycin D (7-AAD) viability dye, phycoerythrin-conjugated anti-insulin and fluorescein-isothiocyanate-conjugated anti-GLUT2 antibodies, and analyzed by flow cytometry to quantify the expression of GLUT2 in insulin-positive beta cells of islets. GLUT2-positive beta cells were gated on 7-AAD-negative and insulin-positive live beta cells. A) Representative flow cytometry plots of GLUT2 staining. B) Percentage of GLUT2-positive, insulin-positive beta cells. n = 6 for each group. *p < .05. **p < .01. Data are expressed as mean ± SEM.

The glucose transporter GLUT2 is responsible for glucose sensing and uptake in beta cells of porcine pancreatic islets [35]. Islets cultured in media supplemented with 100 μM Nec-1 (49.2 ± 2.8%) had a significantly enhanced GLUT2 expression in beta cells compared to islets cultured in control media (29.8 ± 1.8%) or media supplemented with 25 and 50 μM Nec-1 on day 7 of tissue culture (26.1 ± 4.4% and 33.5 ± 5.4%, respectively) (p < .01, p < .01, and p < .05, respectively, Fig 7A and 7B). The GLUT2 expression in beta cells of islets cultured in media added with 200 μM Nec-1 (39.1 ± 3.2%) was similar to islets cultured in either control media or media supplemented with 25, 50, and 100 μM Nec-1 (p = .42, p = .13, p = .83, and p = .34, respectively, Fig 7A and 7B).

### 100 μM Nec-1 augments the insulin secretion of PPIs in response to glucose challenge

**Fig 8:**
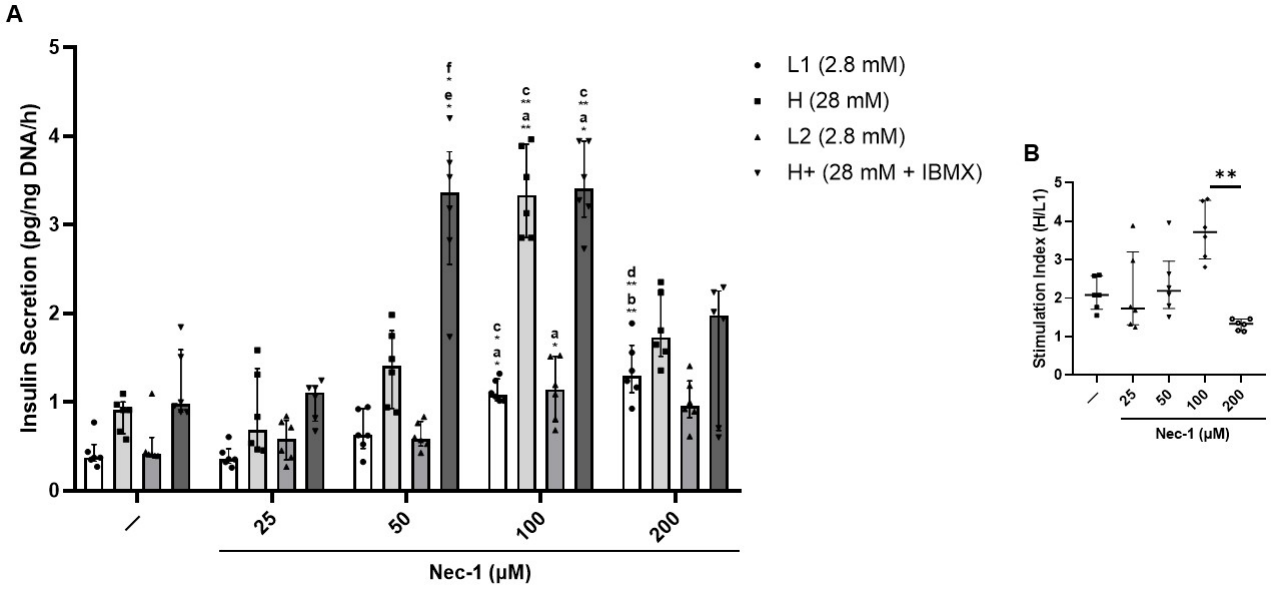
Glucose-stimulated insulin secretion in islets after 7 days of tissue culture in control media (—) or media supplemented with 4 different doses of necrostatin-1 (25, 50, 100, or 200 μM of Nec-1) on day 3. 100 islet equivalents were incubated at 37°C and 5% CO^2^ in the following order of glucose media for 1 hour each: 2.8mM (L1), 28mM (H), 2.8mM (L2), and 28mM + 0.1mM IBMX (H+) glucose media. The insulin concentration in each media was measured using ELISA and normalized to the DNA content of each islet sample. Glucose-induced insulin stimulation index was calculated as the ratio of insulin content in H media over L1 media. A) Insulin secretion per hour in response to glucose challenge. B) Islet glucose-induced insulin stimulation index. n = 6 for each group. a.—vs 100. b.—vs 200. c. 25 vs 100. d. 25 vs 200. e.—vs 50. f. 25 vs 50. *p < .05. **p < .01. Data are expressed as median and interquartile range.

On day 7 of tissue culture, islets underwent incubation in glucose media of the following concentrations for one hour each—2.8mM (L1), 28mM (H), 2.8mM (L2), and 28mM + 0.1mM IBMX (H+)—to assess insulin secretion capacity in response to glucose challenge. In comparison to control islets cultured in media without Nec-1 (L1: .37 pg/ng DNA/h [.33, .52], H: .79 pg/ng DNA/h [.62, .93], L2: .41 pg/ng DNA/h [.40, .60], and H+: .98 pg/ng DNA/h [.89, 1.6]), islets cultured in 25 μM Nec-1 supplemented media (L1: .36 pg/ng DNA/h [.31, .47], H: .68 pg/ng DNA/h [.46, 1.4], L2: .58 pg/ng DNA/h [.35, .79], and H+: 1.1 pg/ng DNA/h [.78, 1.2]) showed no significant differences in the amount of secreted insulin (L1: p = .99, H: p = .99, L2: p = .99, and H+: p = .99, Fig 8A). Culturing islets in media added with Nec-1 at 50 μM (L1: .63 pg/ng DNA/h [.48, .93], H: 1.4 pg/ng DNA/h [.93, 1.8], L2: .59 pg/ng DNA/h [.50, .78], and H+: 3.4 pg/ng DNA/h [2.6, 3.8]) could only increase the insulin secretion in H+ glucose media, but not L1, H, or L2 glucose media, compared to control islets and islets cultured in 25 μM Nec-1 supplemented media (L1: p = .99, .99, H: p = .99, .99, L2: p = .99, .99, and H+: p < .05, < .05, respectively, Fig 8A). After culturing in media added with 100 μM Nec-1 (L1: 1.1 pg/ng DNA/h [1.0, 1.3], H: 3.3 pg/ng DNA/h [2.9, 3.9], L2: 1.1 pg/ng DNA/h [.78, 1.5], and H+: 3.4 pg/ng DNA/h [3.1, 3.9]), the insulin secretion of these islets in L1, H, L2, and H+ glucose media was significantly higher than control islets, respectively (p < .05 for all four different glucose media, Fig 8A). Moreover, islets cultured in media added with 100 μM Nec-1 had a significantly enhanced insulin-releasing capacity after incubation in L1, H, and H+ glucose media compared to islets cultured in 25 μM Nec-1 supplemented media (p < .05 for all three different glucose media, Fig 8A).

Compared to control islets on day 7 of tissue culture, culturing islets in media supplemented with 200 μM Nec-1 (L1: 1.3 pg/ng DNA/h [1.1, 1.6], H: 1.7 pg/ng DNA/h [1.5, 2.3], L2: .96 pg/ng DNA/h [.82, 1.2], and H+: 1.9 pg/ng DNA/h [.68, 2.3]) could only significantly raise the insulin secretion in L1 glucose media, but not in H, L2, or H+ glucose media (L1: p < .01, H: p = .17, L2: p = .07, and H+: p = .99, Fig 8A). Furthermore, increasing the dose of Nec-1 to 200 μM did not improve the insulin secretion in all four different glucose media but resulted in a significantly lower glucose-induced insulin stimulation index (1.3 [1.1, 1.5]) when compared to culturing islets in media supplemented with Nec-1 at 100 μM (3.7 [3.0, 4.5]) (L1: p = .99, H: p = .99, L2: p = .99, H+: p = .14, and SI: p < .01, Fig 8A and 8B).

## Discussion

Islet allotransplantation has offered T1DM patients a potentially curative treatment [7, 36]. Recently, T1DM patients who suffered from severe hypoglycemia despite the best medical treatment have been shown to have improved glycemic control, reduced severe hypoglycemic events, and enhanced awareness of hypoglycemia after islet allotransplantation in a phase 3 trial at eight islet transplantation centers in North America [37]. Furthermore, these same subjects reported a marked improvement in condition-specific health-related quality of life and functional health status after islet allotransplantation [38]. Despite proven benefits, the stringent criteria for donor selection and scarcity of donor pancreata have prevented the widespread clinical application of islet allotransplantation [9]. In contrast, the use of young porcine islets for clinical islet xenotransplantation could not only offer a cost-effective and scalable alternative, but has also been shown to lessen the need for exogenous insulin administration, heighten hypoglycemic awareness, and reduce HbA1C levels in multiple clinical trials [12, 14, 39, 40]. Nevertheless, the prolonged culture period required for the functional maturation of young porcine islets is associated with a substantial islet loss [16, 19]. To facilitate their clinical use, the tissue culture media must be optimized to preserve islet quantity and improve islet quality before transplantation.

As young porcine islets largely consist of immature endocrine cells and pancreatic progenitor cells that are capable of differentiation of proliferation during culture and after transplantation, various studies have explored the utilization of cytoprotective agents and growth factors to prevent islet loss and enhance islet development [17–19, 41]. Previous studies have identified that Nec-1, a potent inhibitor of necroptosis, could reduce islet cell death and DAMP release after exposure to nitric oxide or culture in hypoxic conditions [28, 29]. Recently, the supplementation of Nec-1 to the tissue culture media of PPIs has been reported to mitigate islet loss, improve the development of endocrine cells, and augment insulin secretion [17, 30]. However, the interpretation of the novel effects of Nec-1 on islets is hindered by the use of only one same dose of Nec-1 in all these studies since Nec-1 has been demonstrated to have varying activity levels based on the concentration and type of cells that were used [25, 28, 42–44]. To optimize the dose of Nec-1 and maximize its effects on islets, the present study evaluated the effects that 25, 50, 100, and 200 μM of Nec-1 supplementation on day 3 of tissue culture had on the recovery, viability, endocrine cellular composition, GLUT2 expression in beta cells, and glucose-stimulated insulin secretion of PPIs on day 7 of tissue culture. While the viability of both intact islets and dissociated islet cells were unaffected by Nec-1 regardless of its concentration, the supplementation of 100 μM Nec-1 to islet tissue culture media on day 3 was the most effective dose to improve islet recovery, proportion of endocrine cells, GLUT2 expression in beta cells, and insulin secretion in response to glucose challenge after 7 days of tissue culture.

The current data showing a reduction in islet recovery after 7 days of culture in media without Nec-1 supplementation is in accordance with past studies, in which a substantial loss of young porcine islets could be observed throughout the prolonged in vitro tissue culture period [16, 19]. In support of our results that lower doses of Nec-1 at 25 and 50 μM could not improve the recovery of PPIs on day 7 of tissue culture compared to control islets, previous findings have demonstrated that Nec-1 treatment on rat cardiomyocytes at a low dose of 30 μM failed to delay the opening of mitochondrial permeability transition pores while 100 μM Nec-1 treatment could significantly prolong the time to pore opening [43]. Similarly, Nec-1 treatment at 100 μM was more potent than 30 μM at inhibiting the kinase activity of RIP1 in TNFα-treated FADD-deficient Jurkat cells and Sf9 cells [44]. In another study that incubated murine βTC-6 insulinoma cells in media added with GNSO, Nec-1 treatment at 30 μM was significantly less effective than 100 μM at reducing the amount of cell death [28]. Wu et al. have reported that increasing the optimal concentration of Nec-1 by two times caused a decrease in the effects of Nec-1 to reverse the death of 6-hydroxydopamine treated PC12 cells [42]. The current results agree with this finding as the protective effects of Nec-1 to enhance the recovery of PPIs after 7 days of tissue culture were lost when the optimal dose was doubled to 200 μM on day 3. Our identification of the optimal dose of Nec-1 to be 100 μM is also supported by prior Nec-1-based studies on islets [28, 29]. The treatment of Nec-1 at 100 μM has been demonstrated by Paredes-Juarez et al. to preserve the nuclear DNA content of human islets after 7 days of culture in low-nutrient media at 1% oxygen level [29]. Tamura et al. have also reported that 100 μM Nec-1 treatment of isolated murine islets significantly ameliorated GNSO-induced islet cell death [28]. The present findings of unchanged islet viability measured by two assays after culturing PPIs in Nec-1 supplemented media for 7 days during optimal culture conditions are concordant with previous evidence, indicating that Nec-1 did not affect the survival of both murine and human islets when incubated at standard islet culture conditions [28, 29]. Another plausible explanation for the unaltered islet viability in these studies is that changes in islet death due to necroptosis are exceedingly difficult to detect. In HIV-1-infected CD4+ T lymphocytes, 7-AAD and annexin-V co-staining was utilized to exclude apoptotic cells and identify that Nec-1 treatment significantly reduced necroptosis even though the difference in cell death between untreated and Nec-1 treated cells was below 1% [45]. Taken together, these findings confirm the use of 100 μM Nec-1 as a supplement to enhance islet culture media.

During the process of pancreas procurement, organ preservation, islet isolation, and prolonged *in vitro* tissue culture, islets withstand substantial ischemic damages and upregulate various pro-inflammatory mediators [46–50]. One study has noted that the expression of anti-apoptotic B-cell lymphoma 2 and tissue factor mRNA was lowest and highest on day 6 of tissue culture, respectively, suggesting that the islet inflammatory response peaks during the first week post isolation [16]. Besides impairing cell survival, islet inflammation has been linked to the dedifferentiation of beta- and alpha cells [51–54]. As islet inflammation and DAMP release have been shown to be inhibited by Nec-1 treatment, the enhanced proportion of endocrine cells after Nec-1 supplementation could be due to the activity of Nec-1 to block islet inflammatory response and allow islet development during the first week of tissue culture [28, 29]. Intriguingly, raising the dose of Nec-1 to 200 μM abolished the effects of Nec-1 to expand islet endocrine cellular composition. A previous study has similarly reported that the protective effects of Nec-1 to mitigate 6-hydroxydopamine-induced cell death were lost after doubling the dose of Nec-1 from its optimal concentration [42]. Moreover, increasing the optimal dose of Nec-1 by three times resulted in cell toxicity and further decreased cell survival to an even lower level than 6-hydroxydopamine-only treated cells [42]. Since the mass of endocrine cells has been correlated to islet function, the unchanged insulin secretion of islets cultured in media supplemented with 25, 50, and 200 μM Nec-1 compared to control islets may partly be because of the nonsignificant increase in the endocrine cellular content of these islets on day 7 of tissue culture [17, 18, 55]. For instance, Hassouna et al. have reported that young porcine islets with a lower proportion of endocrine cells secrete significantly less insulin than those with a more developed proportion of endocrine cells after 20 days of stepwise culture in differentiation media [18]. The inferior insulin secretion capacity of islets cultured in media added with 25, 50, and 200 μM Nec-1 could also be due to the diminished GLUT2 expression in beta cells compared to islets cultured in 100 μM Nec-1 supplemented media. These findings are in line with a previous study, in which a higher GLUT2 expression in young porcine islets incubated in media added with controlled-release superoxide dismutase-embedded poly(lactide-co-glycolide) microspheres led to superior insulin release during glucose challenge [56]. The significant increase in both endocrine cellular composition and GLUT2 expression in beta cells of islets cultured in media supplemented with 100 μM Nec-1 is most likely why these islets had the highest insulin secretion capacity. Furthermore, adding Nec-1 at a higher dose than the optimal concentration has been demonstrated to damage explained murine outer hair cells in culture, worsen the viability of PC12 cells after incubation in 6-hydroxydopamine, and enhance infarct size in isolated Langendorff perfused mouse hearts [26, 42, 43]. Our findings and previous studies done by others suggest that supplementing Nec-1 under or over its optimal dose may not only be ineffective, but can also produce toxic actions.

In conclusion, the present study, to our best knowledge, is the first to examine the impacts of day-3 Nec-1 supplementation at several different doses—0, 25, 50, 100, and 200 μM on PPIs after 7 days of in vitro tissue culture. The addition of 100 μM Nec-1 to the culture media of PPIs was more effective than 25, 50, and 200 μM Nec-1 at mitigating islet loss, increasing the proportion of endocrine cells, upregulating GLUT2 expression in beta cells, and augmenting insulin secretion in response to glucose challenge. Besides demonstrating the dose-dependent effects of Nec-1 on young porcine islets during tissue culture, our findings also support previous evidence of Nec-1 toxicity at high concentrations. Future studies exploring the use of novel growth factors on young porcine islets should consider determining the optimal doses to maximize their efficacy in facilitating islet maturation and function after transplantation.
